# Effect of Ylang-Ylang (*Cananga odorata* Hook. F. & Thomson) Essential Oil on Acute Inflammatory Response In Vitro and In Vivo

**DOI:** 10.3390/molecules27123666

**Published:** 2022-06-07

**Authors:** Robson Araújo de Freitas Junior, Paloma Kênia de Moraes Berenguel Lossavaro, Cândida Aparecida Leite Kassuya, Edgar Julian Paredes-Gamero, Nelson Carvalho Farias Júnior, Maria Inês Lenz Souza, Francielli Maria de Souza Silva-Comar, Roberto Kenji Nakamura Cuman, Denise Brentan Silva, Mônica Cristina Toffoli-Kadri, Saulo Euclides Silva-Filho

**Affiliations:** 1 Pharmaceutical Sciences, Food and Nutrition College, Federal University of Mato Grosso do Sul, Campo Grande 79070-900, Brazil; araujo.r@outlook.com.br (R.A.d.F.J.); paloma.lossavaro01@gmail.com (P.K.d.M.B.L.); edgar.gamero@ufms.br (E.J.P.-G.); denise.brentan@ufms.br (D.B.S.); monica.kadri@ufms.br (M.C.T.-K.); 2 Health Sciences College, Federal University of Grande Dourados, Dourados 79825-900, Brazil; candida2005@gmail.com (C.A.L.K.); nelsonjunior@ufgd.edu.br (N.C.F.J.); 3 Biosciences Institute, Federal University of Mato Grosso do Sul, Campo Grande 79070-900, Brazil; maria.souza@ufms.br; 4 Department of Pharmacology and Therapeutics, State University of Maringá, Maringá 87020-900, Brazil; franciellimss@gmail.com (F.M.d.S.S.-C.); rkncuman@uem.br (R.K.N.C.)

**Keywords:** ylang-ylang, essential oil, inflammatory response, natural products

## Abstract

The aim of this study is to evaluate the phytochemical profile, oral acute toxicity, and the effect of ylang-ylang (*Cananga odorata* Hook. F. & Thomson) essential oil (YEO) on acute inflammation. YEO was analyzed by gas chromatography/mass spectrometry. For in vitro tests, YEO was assessed using cytotoxicity, neutrophil chemotaxis induced by *N*-formyl methionyl leucyl phenylalanine (fMLP), and phagocytic activity tests. YEO was orally administered in zymosan-induced peritonitis, carrageenan-induced leukocyte rolling, and adhesion events in the in situ microcirculation model and in carrageenan-induced paw edema models. YEO (2000 mg/kg) was also tested using an acute toxicity test in Swiss mice. YEO showed a predominance of benzyl acetate, linalool, benzyl benzoate, and methyl benzoate. YEO did not present in vitro cytotoxicity. YEO reduced the in vitro neutrophil chemotaxis induced by fMLP and reduced the phagocytic activity. The oral treatment with YEO reduced the leukocyte recruitment and nitric oxide production in the zymosan-induced peritonitis model, reduced rolling and adherent leukocyte number induced by carrageenan in the in situ microcirculation model, and reduced carrageenan-induced edema and mechanical hyperalgesia. YEO did not present signs of toxicity in the acute toxicity test. In conclusion, YEO affected the leukocyte activation, and presented antiedematogenic, anti-hyperalgesic, and anti-inflammatory properties.

## 1. Introduction

Aromatic plants and essential oils are widely used for various purposes, such as food preparation and preservation, and cosmetic products, in addition to being used for medicinal purposes since they have relevant biological activities [1]. The pharmacological properties of medicinal plants are attributed, partially, to the presence of essential oils and their constituents [2]. Essential oils are secondary metabolites produced by various aromatic plants, consisting basically of monoterpenes, sesquiterpenes, and phenylpropanoids [3,4]. Essential oils exhibit, among others, antibacterial, anti-oxidative, anti-inflammatory, and cancer chemoprotective activities [3].

Ylang-ylang (*Cananga odorata* Hook. F. & Thomson) is a medicinal plant of the Annonaceae family and can be found natively in Asian countries, and also in countries such as the Philippines, Malaysia, Indonesia, and Madagascar. Ylang-ylang essential oil (YEO) can be obtained from *C. odorata* flowers and it is widely used in the food, perfume, and aromatherapy industries [5]. Ylang-ylang extracts and YEO have demonstrated, among others, antiviral [6], antimicrobial [7,8], antioxidant [9], and sedative activities [10]. Studies of YEO composition showed the presence of methyl benzoate, geraniol, eugenol, linalool, benzyl acetate, pinene, and caryophyllene [5]. However, the geographical origin, climate, and seasonal variations where the plant is obtained could influence the essential oil composition [11].

Inflammation is an immune response that can be initiated by several factors, such as pathogens, tissue damage, and toxins [12]. This response acts by removing pathogens and promoting repair and tissue homeostasis [13]. The inflammatory response is characterized by redness, edema formation, temperature increase, pain, and tissue function reduction, which result from vascular and cellular events [12,14]. During the inflammatory process, important changes in the microcirculation are observed, such as vascular permeability increase, leukocytes recruitment and migration, and the release of inflammatory mediators [13,15].

Cell migration is crucial in the inflammatory response, and neutrophils are the first cells recruited in acute inflammation [16]. The migration of these cells to the inflammatory sites is very important to the resolution of inflammatory processes, in which phagocytosis is essential for the elimination of microbial pathogens by neutrophils. However, the excessive migration of these cells can damage host tissues [17].

Nonsteroidal anti-inflammatory drugs (NSAIDS) are used in clinical practice and promote various adverse effects, such as gastric damage and cardiovascular and kidney lesions, especially during chronic use [18]. Therefore, the search for natural products with anti-inflammatory activity and less adverse effects is relevant.

Several works have demonstrated the anti-inflammatory activity of extracts of *C. odorata*, and some constituents of YEO [19,20,21,22]. However, studies that evaluated the effects of YEO on leukocyte activation and inflammatory response in experimental models are insufficient. Thus, in this study, we aim to evaluate the YEO effect on leukocyte behavior and inflammatory parameters during acute inflammatory response. The chemical composition and the acute oral toxicity of YEO were also investigated.

## 2. Results

### 2.1. Analysis of YEO

The phytochemical composition of YEO was analyzed by gas chromatography-mass spectrometry (GC-MS). The results of the GC-MS analysis showed a higher relative percentage of the following compounds: benzyl acetate (18.21%), linalool (15.23%), benzyl benzoate (11.39%), geranyl acetate (9.46%), methyl benzoate (7.64%), *p*-methyl anisole (7.38%), *trans*-caryophyllene (5.42%), germacrene D (4.61%), and benzyl salicylate (4.47%).

A complete list of the components and their relative quantity is presented in Table 1, and all the mass spectra and total ion chromatogram are illustrated in the Supplementary Materials (Figures S1–S31).

### 2.2. YEO Did Not Induce In Vitro Cytotoxicity

In the MTT test, the cells were exposed to different concentrations of YEO. At the concentrations of 3, 10, 30, and 90 µg/mL, YEO presented a cell viability of 98, 97, 89, and 91%, respectively, indicating that YEO did not present in vitro cytotoxicity.

### 2.3. YEO Reduces Neutrophil Chemotaxis In Vitro

**Figure 1:**
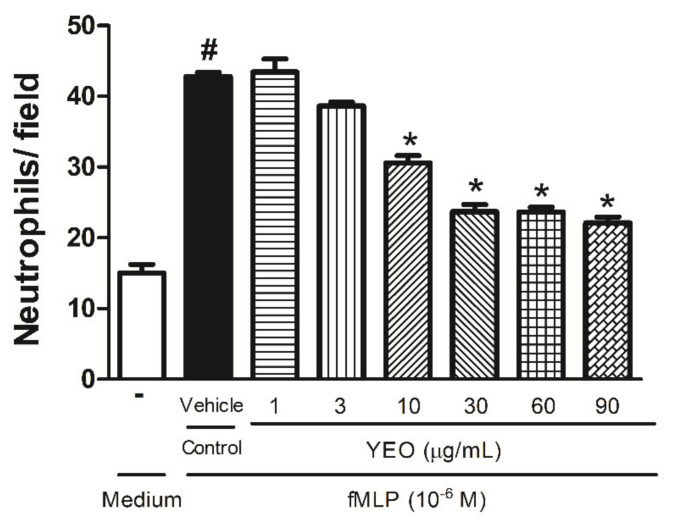
Effect of YEO on in vitro neutrophil chemotaxis. Neutrophils were stimulated with fMLP (10^−^^6^ M) 30 min after YEO treatments at the concentrations of 1, 3, 10, 30, 60, and 90 µg/mL. The results are representative of three independent experiments. ^#^ *p* < 0.05 compared to medium, * *p* < 0.05 compared to the control group (ANOVA, Newman–Keuls test).

In the in vitro chemotaxis assay, fMLP induced considerable neutrophil migration (42.8 ± 1.32 cells/field), compared to the neutrophils exposed to the RPMI-1640 medium (15.03 ± 1.2 cells/field). YEO significantly reduced neutrophil chemotaxis induced by fMLP (10^−6^ M) at the concentrations of 10, 30, 60, and 90 µg/mL (28.6, 44.6, 44.7, and 48.3%, respectively), compared to the control group (Figure 1). The neutrophils exposed to YEO at the concentrations of 1 and 3 µg/mL did not present the significant chemotaxis reduction, compared to the control group.

### 2.4. YEO Reduces Neutrophil Phagocytic Activity

**Figure 2:**
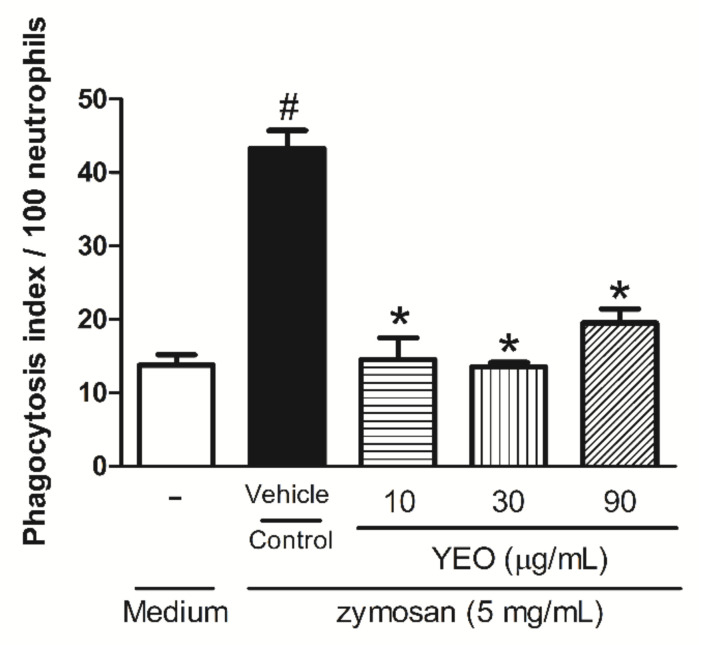
Effect of YEO on the phagocytic activity of neutrophils. Neutrophils were treated with different concentrations of YEO (10, 30, and 90 µg/mL). The results are representative of three independent experiments. ^#^ *p* < 0.05 compared to medium, * *p* < 0.05, compared to the control group (ANOVA, Newman–Keuls test).

The influence of YEO treatment on in vitro phagocytosis is demonstrated in Figure 2. The data showed that the neutrophils exposed to YEO treatment at the concentrations of 10, 30, and 90 µg/mL were significantly reduced in the phagocytosis of zymosan particles by 66.4, 68.7 and 54.9%, respectively, compared to the control group (Figure 2).

### 2.5. YEO Reduces Leukocyte Recruitment and Nitric Oxide Production in the Zymosan-Induced Peritonitis Model

**Figure 3:**
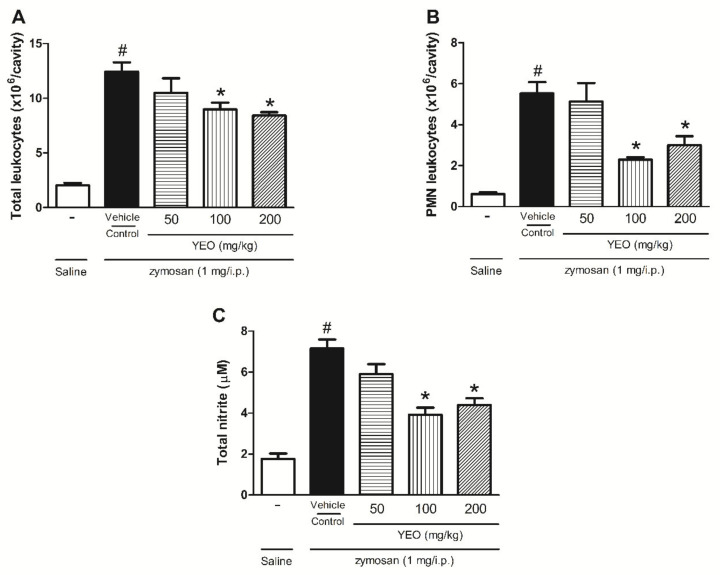
Effect of YEO treatment on migrated leukocyte number and NO production in the peritoneal cavity of Swiss mice 6 h after the zymosan injection (1 mg/cavity/i.p). The effect of YEO treatments on leukocyte counts 6 h after the zymosan injection in Swiss mice (**A**), on PMN number (**B**), and on NO production (**C**). ^#^ *p* < 0.05 compared to saline (vehicle). * *p* < 0.05 compared to the control group (ANOVA, Newman–Keul test).

Six hours after the zymosan injection into mice’s peritoneal cavity, an increase in the leukocyte recruitment (12.45 ± 2.02 × 10^6^ cells/cavity) was observed compared with the control group (saline) (2.08 ± 0.48 × 10^6^ cells/cavity). The YEO treatment at the doses of 100 and 200 mg/kg significantly reduced the total leukocyte number (27.88, and 32.37%, respectively) compared with the control group (Figure 3A). In the leukocyte differential count, it was observed that the pretreatment of animals with YEO at the doses of 100 and 200 mg/kg significantly reduced PMN leukocyte recruitment (58.58 and 45.93%, respectively) (Figure 3B). Nitrite production was used as an indirect measure to nitric oxide (NO) level determination in the peritoneal exudate after zymosan-induced inflammation. The zymosan injection significantly increased the nitrite concentration (7.15 ± 0.87 µM) compared with the group that received the saline injection (1.76 ± 0.59 µM). YEO treatment at the doses of 100 and 200 mg/kg significantly reduced nitrite levels (45.3 and 38.6%, respectively) (Figure 3C). At the dose of 50 mg/kg, YEO treatment did not present a significant reduction in the total and PMN leukocyte numbers and nitrite levels, compared to the control group.

### 2.6. YEO Treatment Reduces Leukocyte Rolling and Adhesion

**Figure 4:**
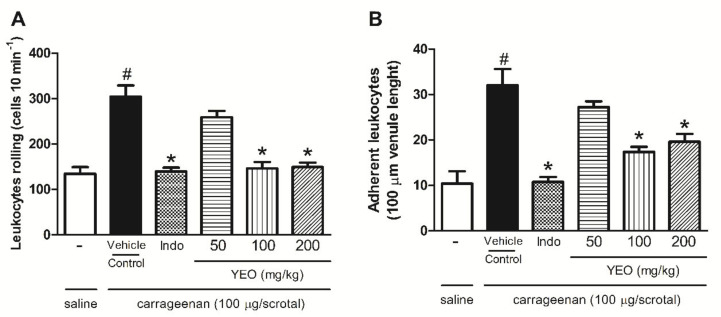
Effect of YEO on leukocyte rolling (**A**) and adhesion (**B**) in the in situ microcirculation test. *Wistar* rats were pretreated with YEO, indomethacin, or the vehicle. After 1 h, saline or carrageenan was injected into the scrotum. The leukocyte behavior was evaluated by intravital microscopy in the spermatic fascia 2 h after the carrageenan injection. ^#^ *p* < 0.05 versus saline. * *p* < 0.05 versus the control group (ANOVA, Newman–Keuls test).

The carrageenan injection (100 µg/cavity) into the scrotum significantly increased the rolling adhesion leukocytes in the endothelium 2 h after stimulation, compared with the control group. YEO treatment at the doses of 100 and 200 mg/kg significantly decreased rolling leukocytes by 51.9 and 50.92%, respectively (Figure 4A), and leukocyte adhesion by 45.6 and 38.75%, respectively (Figure 4B), compared to the control group. Indomethacin, used as reference drug, reduced rolling leukocytes by 54.11% and leukocyte adhesion by 66.4%, compared to the control group.

### 2.7. YEO Treatment Reduces Paw Edema Formation and Mechanical Hyperalgesia Induced by Carrageenan

**Figure 5:**
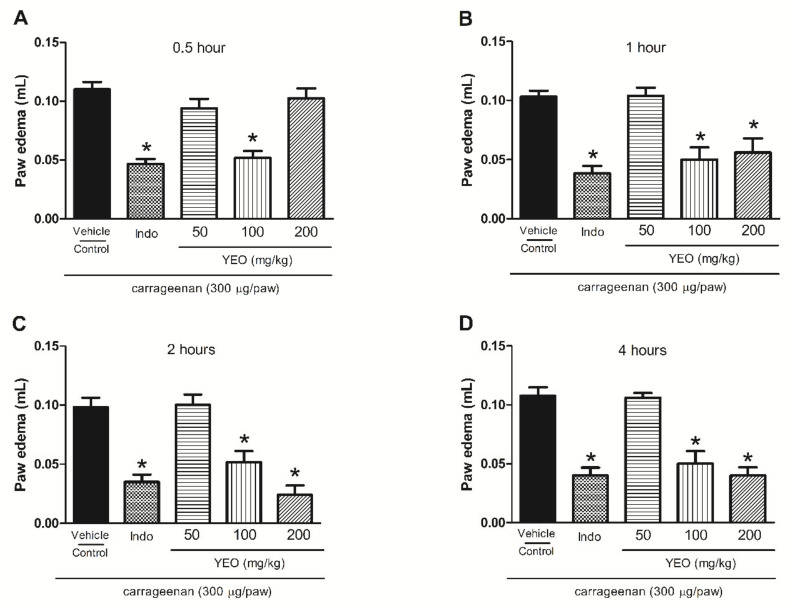
Effect of YEO treatments on carrageenan-induced paw edema in Swiss mice. The figure shows the values at 0.5 (**A**), 1 (**B**), 2 (**C**), and 4 (**D**) h after the edema induction in the control (vehicle), YEO (50, 100 and 200 mg/kg), and indomethacin (5 mg/kg) groups. * *p* < 0.05 compared to the control group (ANOVA, Newman–Keul test).

**Figure 6:**
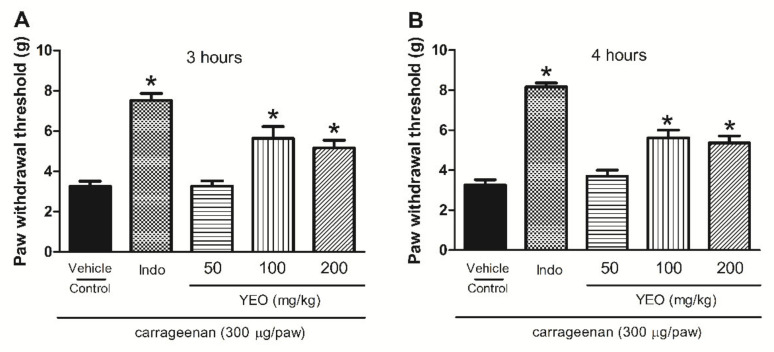
Effect of YEO treatments on carrageenan-induced mechanical hyperalgesia in Swiss mice. The figure shows the values at 3 (**A**) and 4 (**B**) h after the carrageenan injection in the control (vehicle), YEO (50, 100 and 200 mg/kg), and indomethacin (5 mg/kg) groups. * *p* < 0.05 compared to the control group (ANOVA, Newman–Keul test).

In the paw edema and hyperalgesia carrageenan-induced model, the carrageenan intraplantar injection promoted edema formation and hyperalgesia. YEO treatment showed anti-inflammatory activity, promoting the significant reduction in paw edema formation at the doses of 100 and 200 mg/kg (Figure 5). YEO treatment at the dose of 100 mg/kg promoted a reduction in edema formation at all tested times, with a maximum activity at 0.5 h, inhibiting the edema formation by 53.6%, compared to the control group. At the dose of 200 mg/kg, YEO treatment promoted a reduction in edema formation at the times of 1, 2, and 4 h after the carrageenan injection, with a maximum activity at 2 h, reducing the edema formation by 75.5%, compared to the control group (Figure 5A–D). The indomethacin (reference drug) treatment promoted a reduction in paw edema formation in all time-points tested. The YEO treatment at the doses of 100 and 200 mg/kg also demonstrated anti-hyperalgesic activity. The results showed a significant delayed reaction after 3 and 4 h, inhibiting mechanical hyperalgesia (Figure 6A,B). At the dose of 100 mg/kg, YEO treatment reduced mechanical hyperalgesia by 73.53 and 73.14% 3 and 4 h after the carrageenan injection, respectively, compared to the control group. At the dose of 200 mg/kg, YEO treatment reduced mechanical hyperalgesia by 58.76 and 65.43% at the time-points of 3 and 4 h, respectively, compared to the control group. Indomethacin (reference drug) treatment also reduced mechanical hyperalgesia at the time-points of 3 and 4 h after the carrageenan injection, compared to the control group.

### 2.8. YEO Treatment Did Not Induce Acute Oral Toxicity In Vivo

Essential oils and natural products have been widely used by the world’s population. Thus, toxicological screening is very important to ensure safe use. Our data demonstrated that YEO did not cause signs or symptoms of acute and clinical toxicity in the animals. Thus, we can confirm that this essential oil has low toxicity and the Lethal Dose 50 (LD_50_) is above 2000 mg/kg.

## 3. Discussion

In our work, the major constituents found in YEO were benzyl acetate, linalool, benzyl benzoate, and methyl benzoate, which could be relevant to the biological anti-inflammatory activity induced by YEO. This YEO composition is different to that of prior studies; for example, another study with YEO showed the presence of geranyl acetate, cinnamyl and farnesyl acetates, linalool, and geraniol as major constituents [5]. Various factors can influence the phytochemical composition of the essential oils of aromatic plants, such as the method and extraction conditions, the flower condition, geographical factors, and climate and seasonal variations [23]. The present study showed the properties of YEO against in vitro leukocyte chemotaxis, in vitro neutrophil phagocytosis, peritonitis induced by zymosan, leukocyte–endothelium interaction (in situ observation of the scrotal microcirculation), and paw edema and hyperalgesia induced by carrageenan.

Several studies report the effect of essential oils and compounds containing monoterpenes and sesquiterpenes on fMLP-induced leukocyte chemotaxis, with mechanisms involving the participation of pro-inflammatory cytokines [20,24,25,26,27,28,29]. Studies with the RAW264.7 cell culture demonstrated that YEO exhibited in vitro anti-inflammatory activity by the inhibition of the lipoxygenase enzyme [30]. Additionally, the methanolic extract of *Cananga odorata* reduced NO release in the RAW264.7 cell culture [19]. Nevertheless, our work is the first study that demonstrate the effect of YEO on the reduction in in vitro neutrophil chemotaxis induced by fMLP. fMLP is a chemoattractant that induces signal transduction events, leading to several cellular processes, including the diapedesis, chemotaxis, and migration of polymorphonuclear leukocytes (PMN) [31]. Chemotaxis is induced through the production of several pro-inflammatory cytokines, such as interleukin-1β (IL-1β), IL-8, and tumor necrosis factor (TNF), through mitogen-activated protein kinase (MAPK) and phosphatidylinositol 3-kinase (PI-3K) activation, crucial cascades in the development of functional responses and neutrophils in inflammation [32,33]. Linalool, one constituent of YEO, attenuated the production of TNF and IL-6 in cell culture stimulated by lypopolisaccharide (LPS) [34]. Therefore, the presence of linalool in the YEO composition may contribute to the reduction in in vitro cell chemotaxis.

The recruitment and activation of neutrophils are crucial steps in the inflammatory process. The migration of these cells to the site of inflammation/infection is essential for the resolution of inflammation associated with an infectious process [35,36]. Phagocytosis is essential for the microbicidal activity of neutrophils. However, the excessive activation of phagocytes may be related to a higher incidence of tissue damage in inflammatory processes [37,38,39]. YEO reduced leukocyte chemotaxis toward fMLP at the concentrations of 10, 30, 60, and 90 µg/mL. Therefore, we chose the concentrations of 10, 30 and 90 µg/mL to evaluate the effect of YEO on the in vitro phagocytic activity of neutrophils. YEO reduced the phagocytic activity of neutrophils in all the concentrations tested. The YEO effect on the reduction in chemotaxis and phagocytic activity of neutrophils in vitro is not related to cytotoxicity, since YEO did not show cytotoxicity in the MTT test.

The YEO treatment reduced leukocyte recruitment in the zymosan-induced peritonitis model, and the reduction in leukocyte migration was related to the reduction in PMN leukocyte infiltration into the peritoneal cavity. Some works reported the effect of essential oils and terpenes in zymosan-induced peritonitis models [25,29,40]. Zymosan is a polysaccharide component of the cell walls of *Saccharomyces cerevisiae*, widely used in the induction of experimental inflammation, with the consequent production of IL-1, IL-6, TNF, chemokines, and NO [41,42,43]. Kim et al. (2009) demonstrated that linalool, one constituent of YEO, reduced leukocyte infiltration in a ovalbumin-induced lung inflammation model; the proposed mechanism was the inhibition of NF-KB and the reduction in the production of proinflammatory cytokines and NO [44].

During the inflammatory process, NO is produced by inducible nitric oxide synthase (iNOS) from 1-arginine and is an important mediator involved in the regulation and activation of leukocytes [45]. NO is crucial in the pathogenesis of inflammation; the overproduction of this pro-inflammatory mediator promoted cell activation and tissue damage [46]. Our results demonstrated that treatment with YEO reduced leukocyte recruitment, and this mechanism may be related with a decrease in NO levels, such as that observed in the zymosan-induced peritonitis model. However, the others mediators’ inhibition, such as pro-inflammatory cytokines, may be involved in the action mechanism of YEO. However, more studies are needed to confirm this mechanism.

In order to investigate what steps of leukocyte migration are decreased by YEO in the inflammatory site, an in situ microcirculation test was performed. YEO treatment effect on the in situ microcirculation was evaluated in the postcapillary venules of *Wistar* rats’ internal spermatic fascia. Thus, our results showed that YEO treatment reduces leukocyte rolling and adhesion; a similar effect was observed with the indomethacin treatment (reference drug). Carrageenan-induced pro-inflammatory effects in the peritoneal cavities include cellular activation and the release of TNF, IL-6, IL-1β, prostanoids, and NO [47]. These mediators induce the vascular adhesion molecule-1 (VCAM-1), intercellular adhesion molecule-1 (ICAM-1), and selectin expression, culminating with the processes of rolling and leukocyte adhesion [48]. Indomethacin reduces adhesion molecules expression, such as L-selectin and E-selectin, ICAM-1, and VCAM-1 [49,50]. In a similar manner, YEO could also be acting in these mechanisms. Several studies have demonstrated the effect of essential oils, terpenes, and natural products in the reduction in leukocyte rolling and adhesion [20,25,27,51].

In the carrageenan-induced paw model, YEO inhibited edema and mechanical hyperalgesia at the doses of 100 and 200 mg/kg. In the time course curve, the greatest inhibition of paw edema was observed at 0.5 h, at a dose of 100 mg/kg, and at 2 h, at a dose of 200 mg/kg, after carrageenan paw injection, while a significant antinociceptive response (mechanical hyperalgesia) was detected at all the times analyzed, at the doses of 100 and 200 mg/kg. The carrageenan intraplantar injection promotes paw edema, inflammatory cell infiltration, the release of inflammatory mediators, such as IL-1β and IL-6, the increase in cyclooxygenase-2 (COX-2) expression, and prostaglandin E_2_ (PGE_2_) release [52]. Several works have demonstrated the effect of essential oils and terpenes in the inhibition of paw edema and mechanical hyperalgesia induced by carrageenan [26,53,54]. Linalool, a terpene found in YEO, also reduces paw edema in carrageenan-induced inflammation [21]. Was also demonstrated that the ethanolic extract obtained from the *Cananga odorata* fruit reduces paw edema induced by carrageenan in rats [22].

In our study, all the doses of YEO used in the in vivo tests are considered safe. In the acute toxicity assay, there were no deaths or any signs of toxicity observed after the administration of YEO, having an LD_50_ value above 2000 mg/kg. Although some of the pharmacological properties of YEO have been described in the literature, such as its anxiolytic and antidepressant effects [55,56], this work is the first in the literature to demonstrate the effect of this essential oil on acute inflammatory response. The presence of linalool in the YEO composition could contribute to its anti-inflammatory activity; however, the presence of other compounds could not be discarded.

## 4. Material and Methods

### 4.1. Chemicals and Drugs

YEO, [3-(4,5-dimethylthiazol-2-yl)-2,5-diphenyl-2H-tetrazolium bromide] (MTT), zymosan, ***N***-formyl methionyl leucyl phenylalanine (fMLP), indomethacin, and carrageenan were purchased from Sigma-Aldrich (St Louis, MO, USA). All other chemicals used were of analytical grade.

### 4.2. Chemical Analysis of YEO

YEO was chemically analyzed by gas chromatography/mass spectrometry (GC/MS) using a gas chromatography Shimadzu QP2010 (Shimadzu^®^, Kyoto, Japan) apparatus coupled to a mass spectrometer with an electron ionization (EI) source, which was applied at an ionization energy of 70 eV. The chromatographic column was a Rtx-5MS (30 m × 0.25 mm, 0.25 mm in thickness) using as carrier gas (pressure of 79.7 kPa and column flow rate of 1.30 mL/min). The split ratio was 1:40 and the temperature programming was from 60 to 220 °C and it was increased by 3 °C/min. WILEY 7, NIST 11, and FFNSC databanks were used to identify the constituents, and their retention indices (calculated by the injection of a series of alkanes, from C9 to C22) were compared with those reported by Adams (1995).

### 4.3. Animals

Male and female Swiss mice (weighing 20–30 g) were provided by the Central Animal House of the Federal University of Mato Grosso do Sul (UFMS), and Male *Wistar* rats (180–220 g) were provided by the Central Animal House of the State University of Maringá (UEM). The animals were housed at 22 ± 2 °C under a 12/12 h light/dark cycle. Prior to the experiments, the animals were fasted overnight, with water provided ad libitum. The experimental protocols were approved by the Ethical Committee in Animal Experimentation of the UFMS (protocol number: 1.101/2019), and the Ethical Committee in Animal Experimentation of the UEM (protocol number: 9680050221).

### 4.4. Leukocyte Preparation for In Vitro Assays

To perform the in vitro tests, leukocytes were obtained from the peritoneal cavity of male Swiss mice 4 h after the intraperitoneal zymosan injection (1 mg/animal). To obtain the cells, the mice’s peritoneal cavities were washed with 1 mL of phosphate-buffered saline (PBS) solution containing EDTA. The collected peritoneal exudate was centrifuged (1000 rpm/10 min/4 °C), the supernatant was discarded, and the pellet was resuspended in an RPMI1640 medium containing 0.1% of bovine serum albumin (BSA). The leukocyte viability was verified by the trypan blue method, which was above 98%. Additionally, a differential cell count was performed and 95% of the leukocytes obtained corresponded to neutrophils.

### 4.5. Cell Viability Analysis (MTT Test)

The cell viability assay was performed as previously described [57]. The leukocyte number was adjusted at 5 × 10^5^ cells/well in 100 µL of complete RPMI medium, into 96 well plate. The cells were exposed to YEO at different concentrations (3, 10, 30 or 90 μg/mL) or the vehicle for 90 min. Afterwards, 10 μL of MTT (5 mg/mL) was added to each well and the plate was incubated (37 °C/CO_2_ 5%) for 120 min. After incubation, the supernatant was removed and 100 µL of DMSO was added to each well, and the plate was incubated for 10 min. Subsequently, the absorbance was measured at 540 nm and the cell viability was determined by the formula: Viability cells (%) = (absorbance of treated cells − blank absorbance)/(control absorbance − blank absorbance) × 100. Data were presented as values of three independent experiments performed in triplicate.

### 4.6. In Vitro Neutrophil Chemotaxis

The neutrophil chemotaxis assay was performed as previously described [28]. The neutrophil number (obtained from peritoneal lavage) was adjusted to 1 × 10^6^ cells/mL in an RPMI 1640 medium. The in vitro chemotaxis test was performed using a 48-well microchemotaxis plate (Neuro Probe, Gaithersburg, MD, USA), in which the chambers were separated by a polyvinylpyrrolidone-free polycarbonate membrane (5 µm pore size). *N*-formyl methionyl leucyl phenylalanine (fMLP 10^−6^ M) (used as chemoattractant) and the RPMI 1640 medium were placed in the lower chamber. The neutrophils were exposed to YEO at different concentrations (1, 3, 10, 30, 60, or 90 µg/mL) or the vehicle for 30 min and then placed in the upper chamber. The chambers were incubated (37 °C/CO_2_ 5%) for 1 h. After incubation, the membrane was washed with PBS, and stained with an Instant Prov kit. The membrane area of each well was scored using light microscopy to count the neutrophils in five random fields. The results are expressed as the mean number of neutrophils per field.

### 4.7. In Vitro Phagocytic Activity of Neutrophils

The phagocytic activity of the neutrophil assay was performed as previously described [25]. The neutrophil suspension (2 × 10^6^ cells/mL) was exposed to YEO at different concentrations (10, 30, or 90 μg/mL) or the vehicle for 30 min. To induce phagocytosis, the zymosan particles (5 mg/mL) were opsonized from the incubation with the RMPI medium containing mice plasma (10%) for 30 min. After the neutrophil pretreatment period, the cells were centrifuged, resuspended in 1 mL of the RPMI medium containing 10% of plasma from mice and 10 μL of a zymosan solution (5 mg/mL), and incubated for 30 min (37 °C/CO_2_ 5%). Subsequently, the cells were fixed and stained. The count was held in an optical microscope and the results are expressed as number of neutrophils in phagocytosis by 100 neutrophils.

### 4.8. Leukocyte Recruitment and Nitric Oxide Levels Determination in Zymosan-Induced Peritonitis Model

Male Swiss mice were perorally treated with YEO, at the doses of 50, 100, or 200 mg/kg or vehicle (control group) (*n* = 5–7 animals/ group). After 30 min, the animals received an intraperitoneal zymosan injection (1 mg/cavity) or saline. Six hours later, the mice were euthanatized, and the cells were collected after the washing of the peritoneal cavity with 1 mL of PBS solution containing EDTA. Counts were then performed in total and differential cells. The results are expressed as the leukocytes number per cavity. NO levels were determined from the peritoneal exudate by measuring the nitrite levels by the Griess reaction. The supernatant of the peritoneal exudate (50 mL) was incubated for 10 min with equal volumes of Griess reagent mixtures (1% sulfanilamide in 5% phosphoric acid and 0.1% *N*-1- naphthylethylenediamine dihydrochloride in water). The absorbance was measured at 550 nm. NO concentrations were calculated from a sodium nitrite standard curve. Data are presented as the mm concentration of NO^2−^.

### 4.9. In Situ Intravitral Microscopy Analysis for Rolling and Adhesion Events of Leukocytes in the Microcirculation

YEO effect in the microcirculation was evaluated according to the procedure previously described [27]. Leukocyte rolling and adhesion were evaluated by in situ microcirculation test in the internal spermatic fascia of male *Wistar* rats, 2 h after carrageenan injection (100 µg) into the wall of the scrotal chamber. Thirty minutes before the carrageenan injection, the animals were perorally treated with YEO at the doses of 50, 100, or 200 mg/kg, indomethacin (5 mg/kg, reference drug), or the vehicle (control group) (*n* = 5–7 animals/ group). Two hours after the carrageenan injection, the animals were anesthetized and maintained on a special board thermostatically controlled at 37 °C with a transparent platform for transillumination of the tissue on which the spermatic fascia was exposed and fixed for analysis by microscopy in situ. The preparation was kept moist and warm with a Ringer–Locke solution (pH 7.2). The vessels selected were postcapillary venules with a diameter of 15–25 μm. The number of rolling and adherent leukocytes was determined at 10-min intervals. The leukocytes were considered adherent if they remained stationary for more than 30 s.

### 4.10. Paw Edema and Mechanical Hyperalgesia Induced by Carrageenan

Male Swiss mice were perorally treated with YEO at the doses of 50, 100, and 200 mg/kg, indomethacin (5 mg/kg), or the vehicle (control group) (*n* = 5–7 animals/ group), 1 h before paw edema induction by the intraplantar injection of 100 µL of the carrageenan solution (300 μg/paw) in the right hind paw of all animals; a contralateral paw saline solution 0.9% was injected. The edema was measured at 0.5, 1, 2, and 4 h, and hyperalgesia was evaluated at 3 and 4 h after the carrageenan injection. The paw edema evaluation was made using a plethysmometer (Insight^®^, Riberião Preto, Brazil) [26]. For the evaluation of mechanical hyperalgesia, the animals were placed in a containment box with support for the analgesimeter test for 30 min. Subsequently, for the nociceptive mechanical sensitivity threshold determination, a digital analgesimeter (Von Frey, Insight^®^) [58] was used as a pressure transducer, which records the applied force until the moment of paw withdrawal.

### 4.11. Acute Oral Toxicity Study

The acute oral toxicity test was performed with male and female Swiss mice according to the OECD 425—407 guidelines. YEO (2000 mg/kg) or the vehicle was perorally administered, in a single dose, in male and female animals (*n* = 5 animals/group), fasting for 12 h. All animals remained under observation for 14 days [59,60,61]. The five Hippocratic parameters were observed daily: consciousness, motor coordination, reflexes (auditory and corneal), central nervous system (ataxia, tremors, sedation, and seizure), and autonomic nervous system (piloerection, sialorrhea, cyanosis, ptosis, and tearing) [62], in addition to the determination of body weight and water and food consumption. The animals were euthanized, and organs such as heart, spleen, lungs, liver and kidneys were removed, macroscopically analyzed, and weighed [63].

### 4.12. Statistical Analysis

Data are expressed as the mean ± SEM for each experimental group. The results were statistically analyzed by using a one-way variance analysis (ANOVA), followed by the Newman–Keuls post hoc test. The percentage of inhibition was calculated in relation to the control group. Differences were considered significant when *p* < 0.05.

## 5. Conclusions

In conclusion, YEO presented anti-inflammatory activity. YEO reduces the leukocyte activation during acute inflammatory response. The mechanism of YEO may be related with a decrease in NO levels. The study of acute toxicity indicated that the oral single administration of YEO did not cause any deaths or changes in the general behavior of mice. Further studies are needed to elucidate the mechanism of action of this essential oil on the inflammatory response.

## Figures and Tables

**Table 1:** Chemical composition of *Cananga odorata* essential oil.

Peak	RT (min)	Compound	RI	%
1	4.57	prenyl acetate	921	0.41
2	7.10	*p*-methyl anisole	1019	7.38
3	7.45	1,8-cineole	1030	0.05
4	9.62	methyl benzoate	1096	7.64
5	9.79	linalool	1100	15.23
6	12.28	benzyl acetate	1164	18.12
7	15.99	linalyl acetate	1255	0.21
8	20.91	α-copaene	1374	0.38
9	21.33	geranyl acetate	1384	9.46
10	21.60	β-elemene	1390	0.09
11	22.68	*trans*-caryophyllene	1417	5.42
12	23.82	cinnamyl acetate	1445	6.05
13	24.05	α-humulene	1451	1.80
14	25.01	γ-muurolene	1475	0.42
15	25.17	germacrene D	1479	4.61
16	25.60	prenyl benzoate	1489	0.17
17	25.79	bicyclogermacrene	1494	0.13
18	25.98	α-muurolene	1499	0.21
19	26.12	γ-bisabolene	1502	0.59
20	26.32	α-farnesene	1508	2.02
21	26.49	γ-cadinene	1512	0.22
22	26.87	δ-cadinene	1522	0.98
23	29.12	caryophyllene oxide	1580	0.06
24	31.40	α-cadinol	1641	0.44
25	31.56	α-muurolol	1645	0.10
26	31.86	cadin-4-en-10-ol	1654	0.51
27	34.36	(*z*,*z*)-farnesol	1722	0.38
28	35.80	benzyl benzoate	1764	11.39
29	38.47	farnesyl acetate	1841	0.83
30	39.34	benzyl salicylate	1868	4.47

RT: retention time; RI: retention index on Rtx-5MS.

## Data Availability

All data are available in this publication.

## Supplementary Materials

The following supporting information can be downloaded at: https://www.mdpi.com/article/10.3390/molecules27123666/s1, Figure S1: Total ion chromatogram from *Cananga odorata* essential oil obtained by GC-MS; Figure S2: Mass spectrum of peak 1 (RT = 4.57 min); Figure S3: Mass spectrum of peak 2 (RT = 7.10 min); Figure S4: Mass spectrum of peak 3 (RT = 7.45 min); Figure S5: Mass spectrum of peak 4 (RT = 9.62 min); Figure S6: Mass spectrum of peak 5 (RT = 9.79 min); Figure S7: Mass spectrum of peak 6 (RT = 12.28 min); Figure S8: Mass spectrum of peak 7 (RT = 15.99 min); Figure S9: Mass spectrum of peak 8 (RT = 20.91); Figure S10: Mass spectrum of peak 9 (RT = 21.33 min); Figure S11: Mass spectrum of peak 10 (RT = 21.60 min); Figure S12: Mass spectrum of peak 11 (RT = 22.68 min); Figure S13: Mass spectrum of peak 12 (RT = 23.82 min); Figure S14: Mass spectrum of peak 13 (RT = 24.05 min); Figure S15: Mass spectrum of peak 14 (RT = 25.01 min); Figure S16: Mass spectrum of peak 15 (RT = 25.17 min); Figure S17: Mass spectrum of peak 16 (RT = 25.60 min); Figure S18: Mass spectrum of peak 17 (RT = 25.79 min); Figure S19: Mass spectrum of peak 18 (RT = 25.98 min); Figure S20: Mass spectrum of peak 19 (RT = 26.12 min); Figure S21: Mass spectrum of peak 20 (RT = 26.32 min); Figure S22: Mass spectrum of peak 21 (RT = 26.49 min); Figure S23: Mass spectrum of peak 22 (RT = 26.87 min); Figure S24: Mass spectrum of peak 23 (RT = 29.12 min); Figure S25: Mass spectrum of peak 24 (RT = 31.40 min); Figure S26: Mass spectrum of peak 25 (RT = 31.56 min); Figure S27: Mass spectrum of peak 26 (RT = 31.86 min); Figure S28: Mass spectrum of peak 27 (RT = 34.36 min); Figure S29: Mass spectrum of peak 28 (RT = 35.80 min); Figure S30: Mass spectrum of peak 29 (RT = 38.47 min); Figure S31: Mass spectrum of peak 30 (RT = 39.34 min).

## Sample Availability

Samples of the compounds are available from the authors.
