# Supplementary material for: Effect of Ylang-Ylang (Cananga odorata Hook. F. & Thomson) Essential Oil on Acute Inflammatory Response In Vitro and In Vivo
Source: Molecules. 2022 Jun 7;27(12):3666. doi: 10.3390/molecules27123666 (PMC9231162; doi:10.3390/molecules27123666)
Supplement: Supplementary file 1 [file molecules-27-03666-s001.zip › molecules-1676316-supplementary.pdf]

molecules

MDPI

Supplementary Material

Effect of Ylang-ylang (*Cananga odorata* Hook. F. & Thomson) Essential Oil on Acute Inflammatory Response In Vitro and In Vivo

Robson Araújo de Freitas Junior [1], Paloma Kênia de Moraes Berenguel Lossavaro [1], Cândida Aparecida Leite Kassuya [2], Edgar Julian Paredes-Gamero [1], Nelson Carvalho Farias Júnior [2], Maria Inês Lenz Souza [3], Francielli Maria de Souza Silva-Comar [4], Roberto Kenji Nakamura Cuman [4], Denise Brentan Silva [1], Mônica Cristina Toffoli-Kadri [1] and Saulo Euclides Silva-Filho [1,*]

[1] Pharmaceutical Sciences, Food and Nutrition College, Federal University of Mato Grosso do Sul, Campo Grande 79070-900, Brazil; araujo.r@outlook.com.br (R.A.d.F.J.); paloma.lossavaro01@gmail.com (P.K.d.M.B.L.); edgar.gamero@ufms.br (E.J.P.-G.); denise.brentan@ufms.br (D.B.S.); monica.kadri@ufms.br (M.C.T.-K.)
[2] Health Sciences College, Federal University of Grande Dourados, Dourados 79825-900, Brazil; candida2005@gmail.com (C.A.L.K.); nelsonjunior@ufgd.edu.br (N.C.F.J.)
[3] Biosciences Institute, Federal University of Mato Grosso do Sul, Campo Grande 79070-900, Brazil; maria.souza@ufms.br
[4] Department of Pharmacology and Therapeutics, State University of Maringá, Maringá 87020-900, Brazil; franciellimss@gmail.com (F.M.d.S.S.-C.); rkncuman@uem.br (R.K.N.C.)
* Correspondence: saulo.e@ufms.br

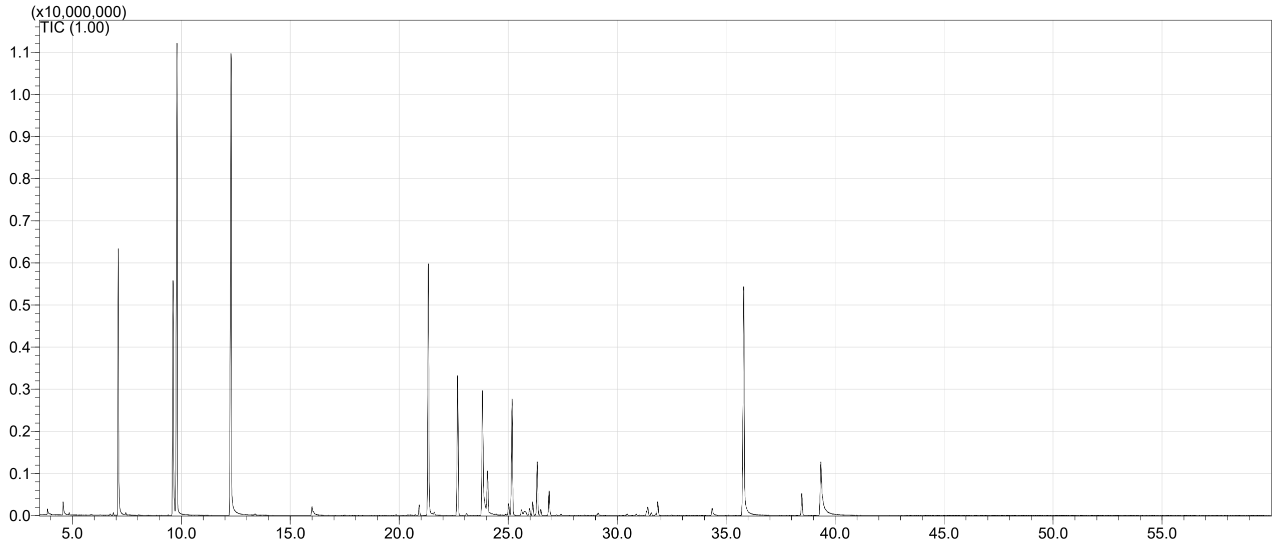

Figure S1. Total ion chromatogram from *Cananga odorata* essential oil obtained by GC-MS.

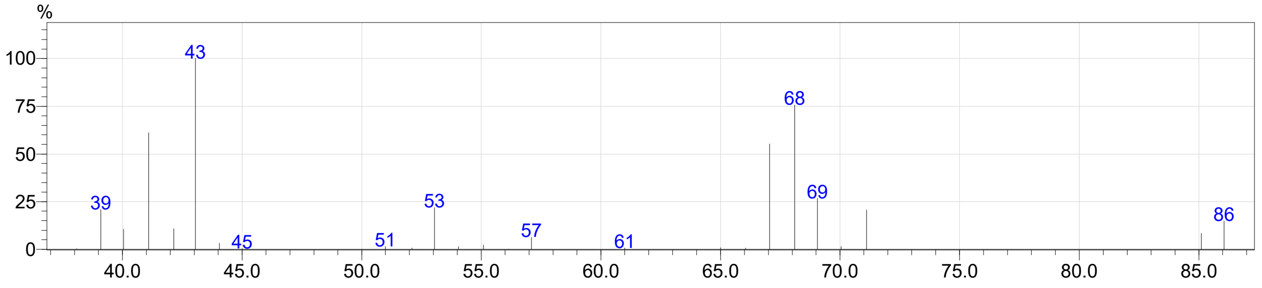

Figure S2. Mass spectrum of peak 1 (RT = 4.57 min).

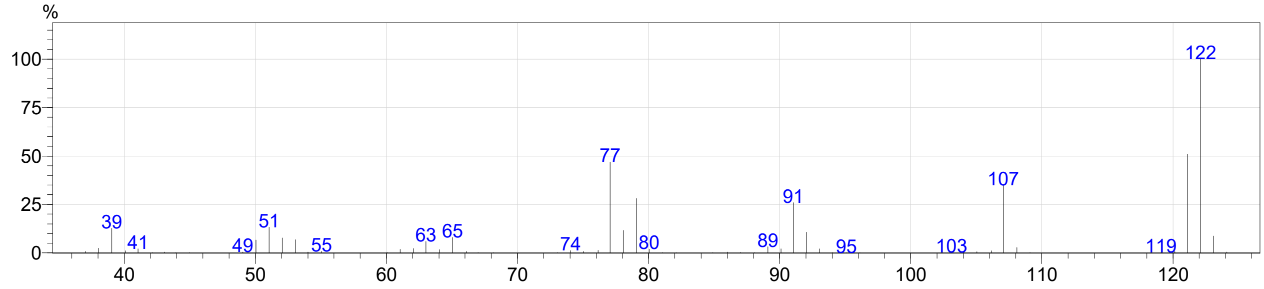

Figure S3. Mass spectrum of peak 2 (RT = 7.10 min).

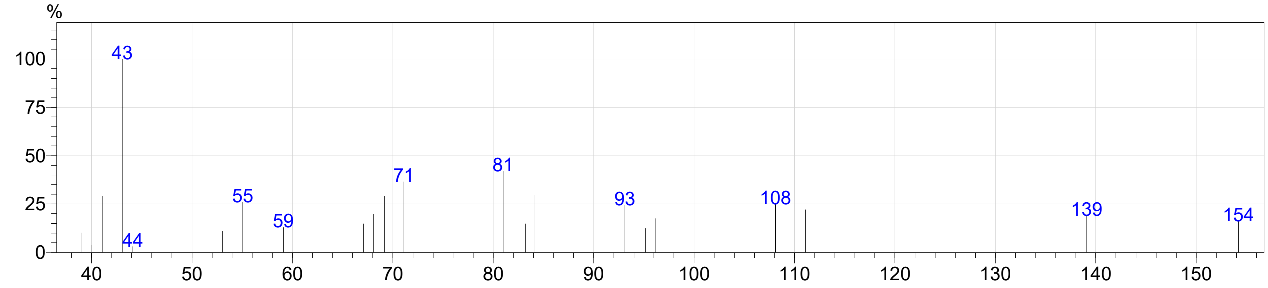

Figure S4. Mass spectrum of peak 3 (RT = 7.45 min).

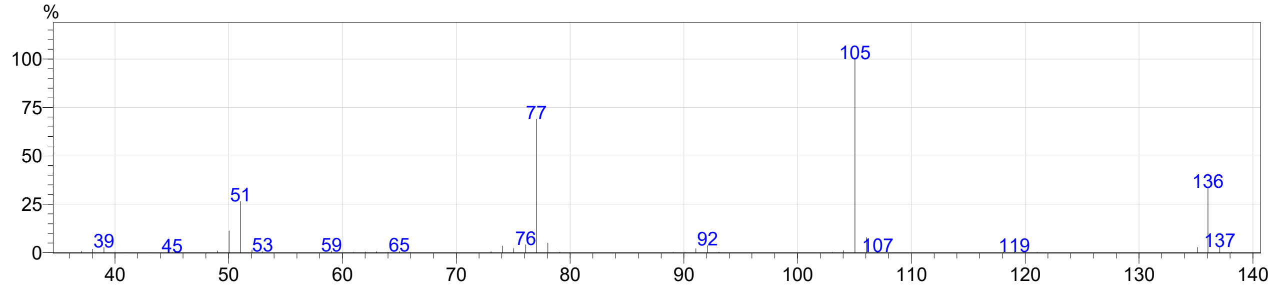

Figure S5. Mass spectrum of peak 4 (RT = 9.62 min).

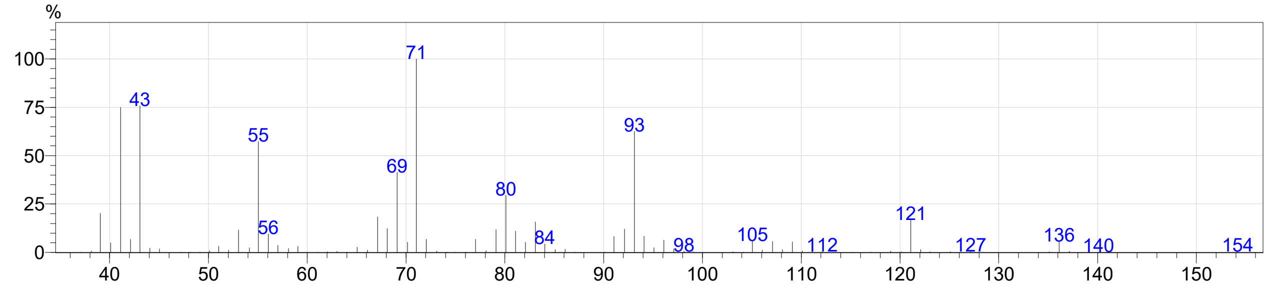

Figure S6. Mass spectrum of peak 5 (RT = 9.79 min).

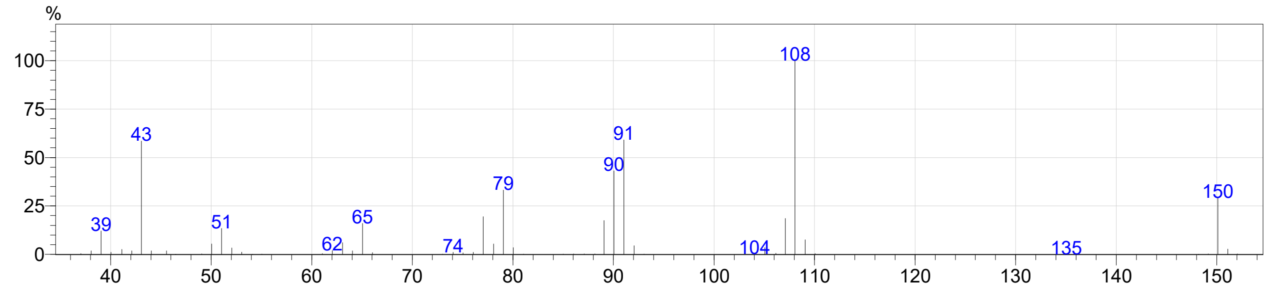

Figure S7. Mass spectrum of peak 6 (RT = 12.28 min).

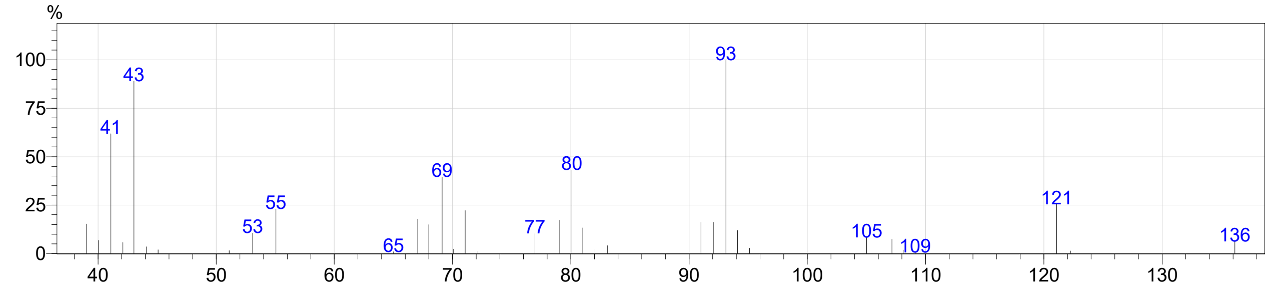

Figure S8. Mass spectrum of peak 7 (RT = 15.99 min).

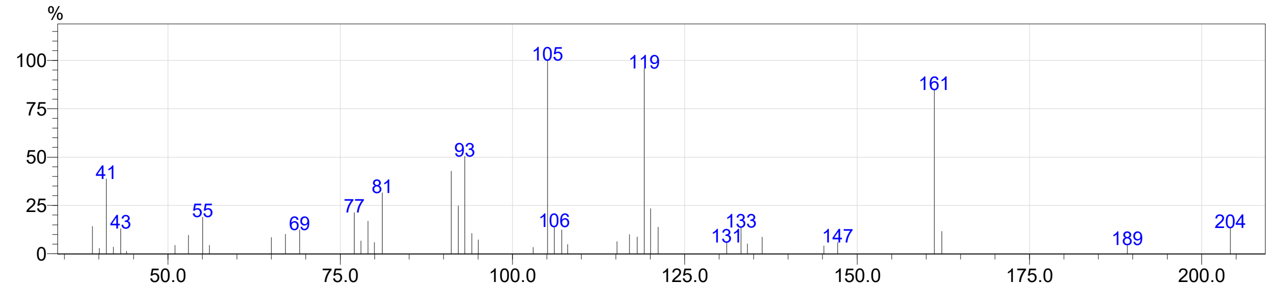

Figure S9. Mass spectrum of peak 8 (RT = 20.91 min).

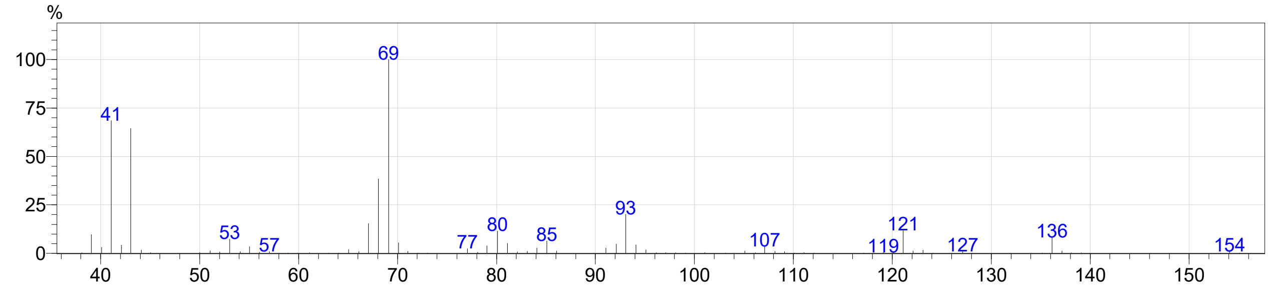

Figure S10. Mass spectrum of peak 9 (RT = 21.33 min).

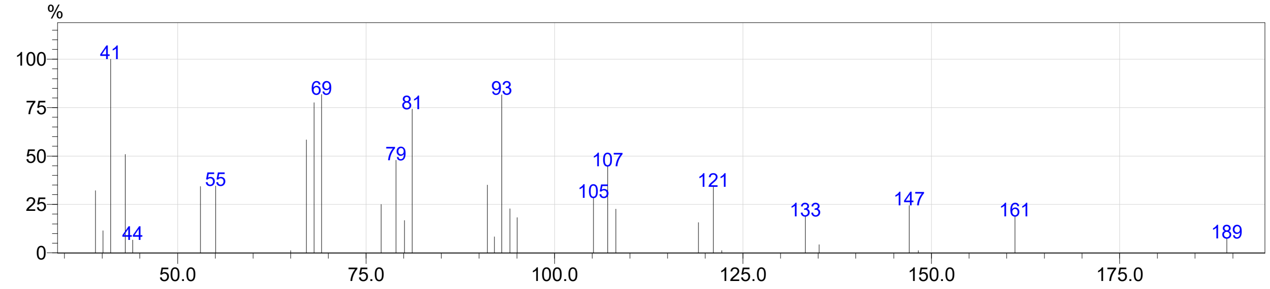

Figure S11. Mass spectrum of peak 10 (RT = 21.60 min).

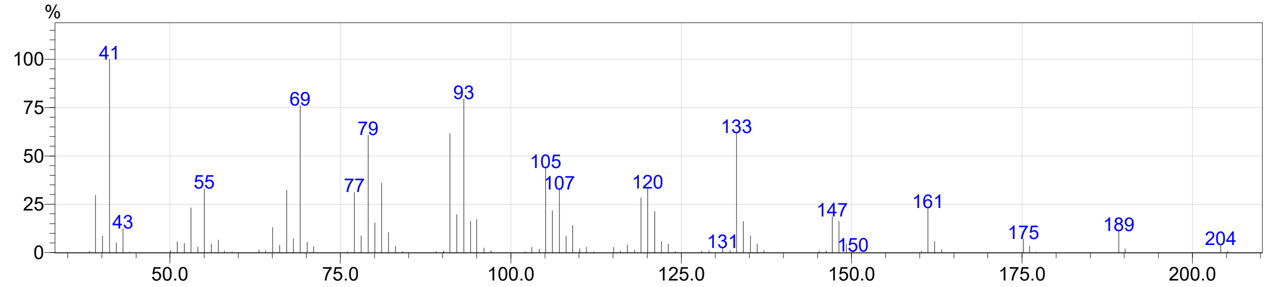

Figure S12. Mass spectrum of peak 11 (RT = 22.68 min).

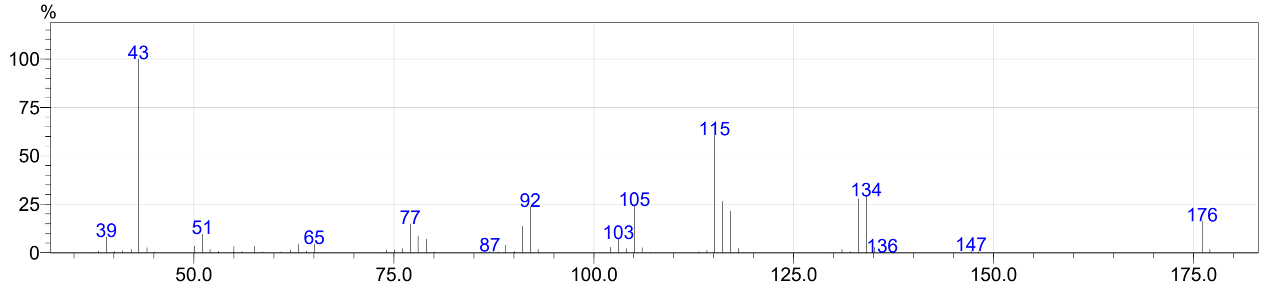

Figure S13. Mass spectrum of peak 12 (RT = 23.82 min).

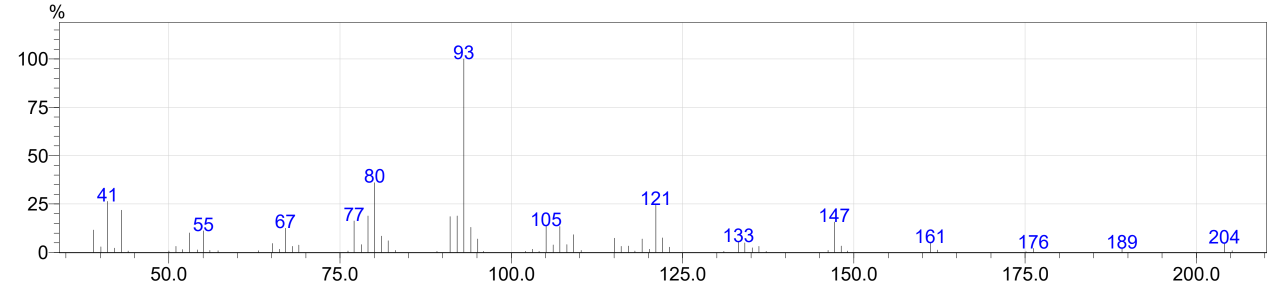

Figure S14. Mass spectrum of peak 13 (RT = 24.05 min).

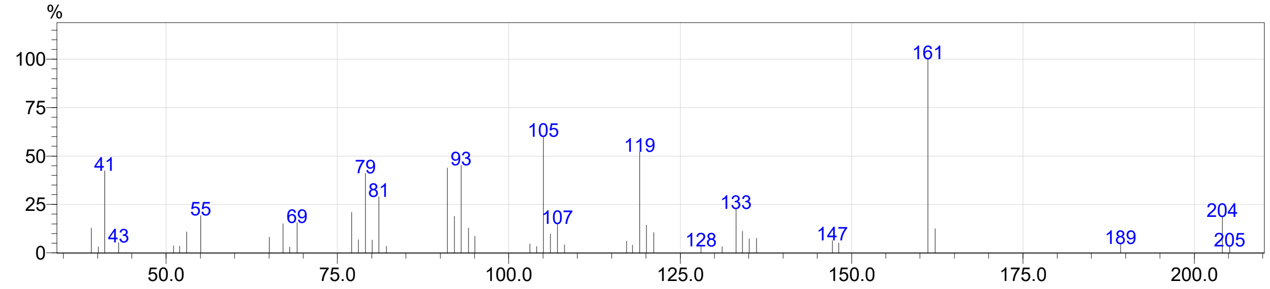

Figure S15. Mass spectrum of peak 14 (RT = 25.01 min).

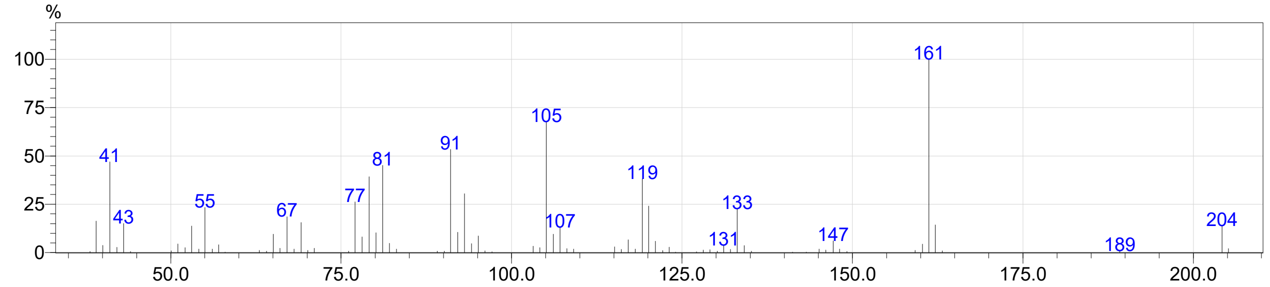

Figure S16. Mass spectrum of peak 15 (RT = 25.17 min).

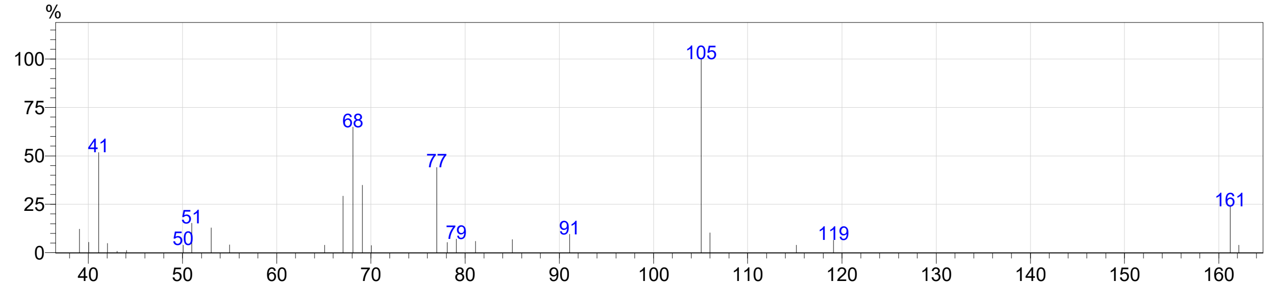

Figure S17. Mass spectrum of peak 16 (RT = 25.60 min).

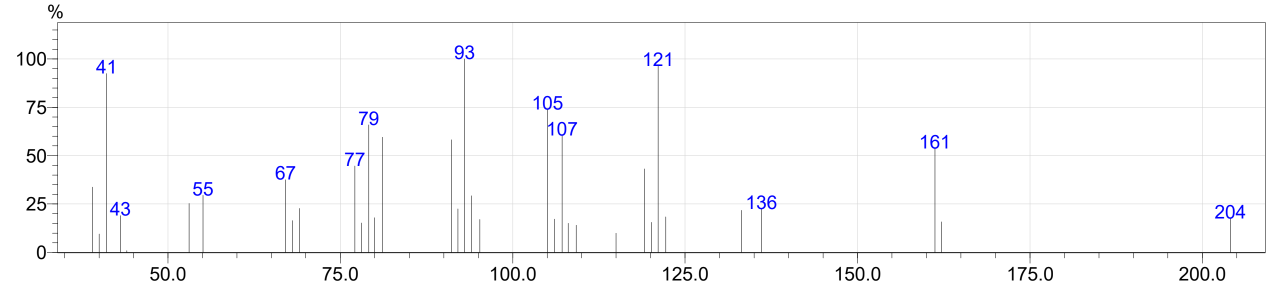

Figure S18. Mass spectrum of peak 17 (RT = 25.79 min).

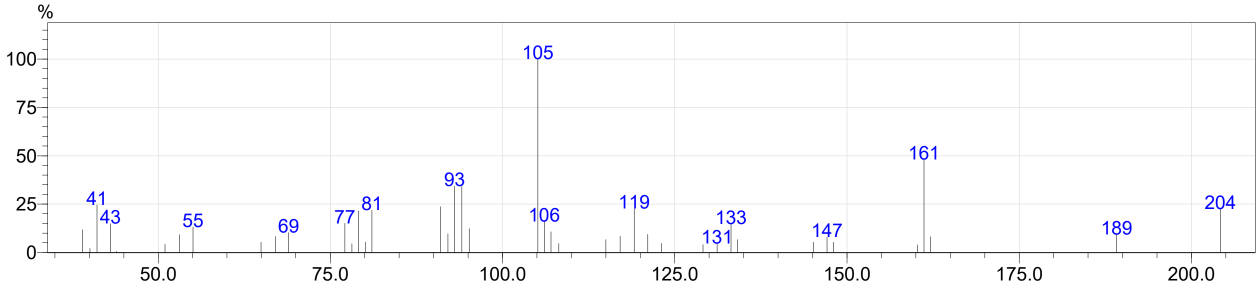

Figure S19. Mass spectrum of peak 18 (RT = 25.98 min).

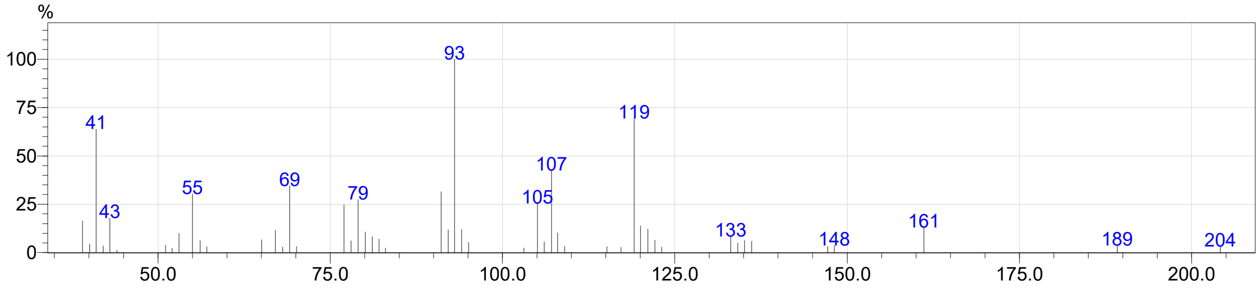

Figure S20. Mass spectrum of peak 19 (RT = 26.12 min).

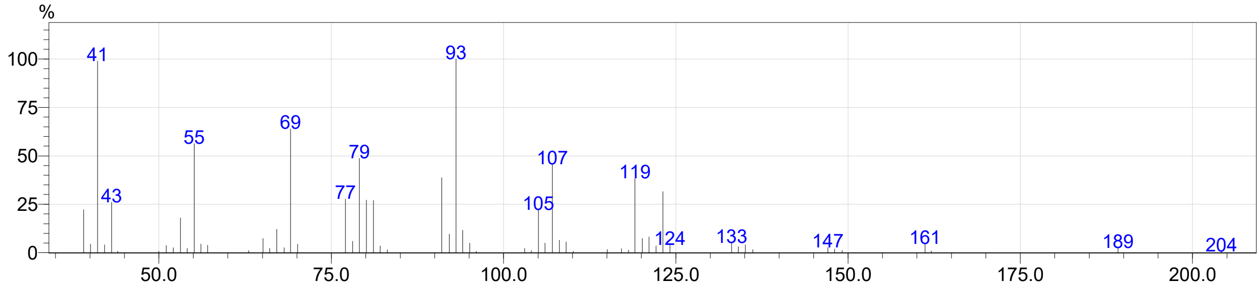

Figure S21. Mass spectrum of peak 20 (RT = 26.32 min).

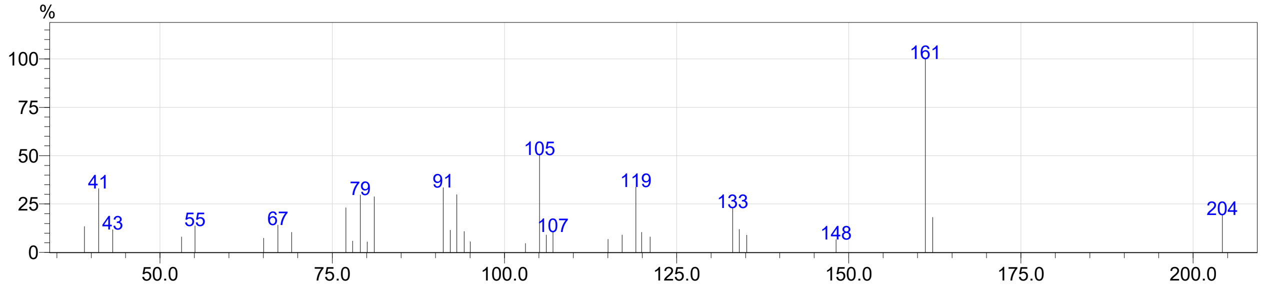

Figure S22. Mass spectrum of peak 21 (RT = 26.49 min).

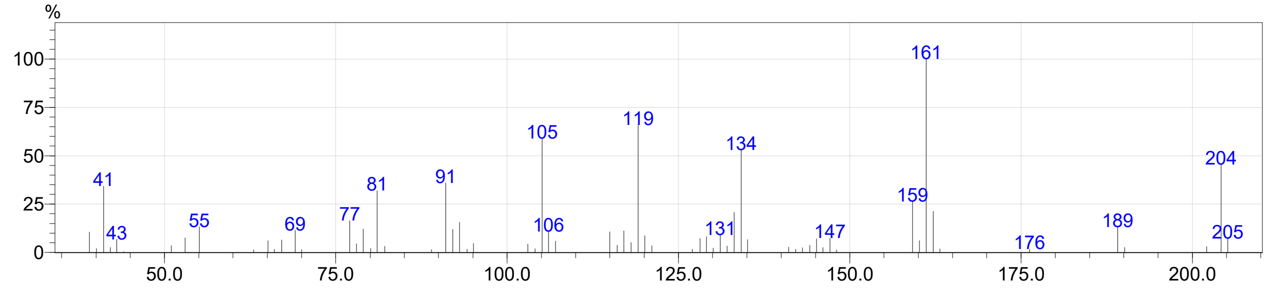

Figure S23. Mass spectrum of peak 22 (RT = 26.87 min).

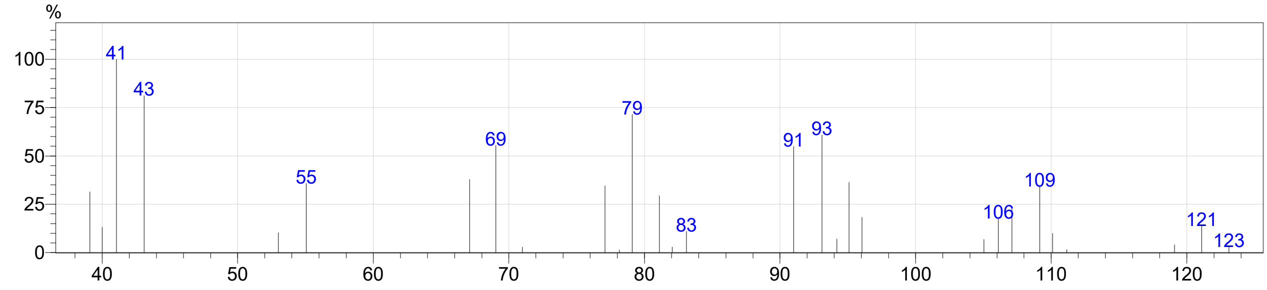

Figure S24. Mass spectrum of peak 23 (RT = 29.12 min).

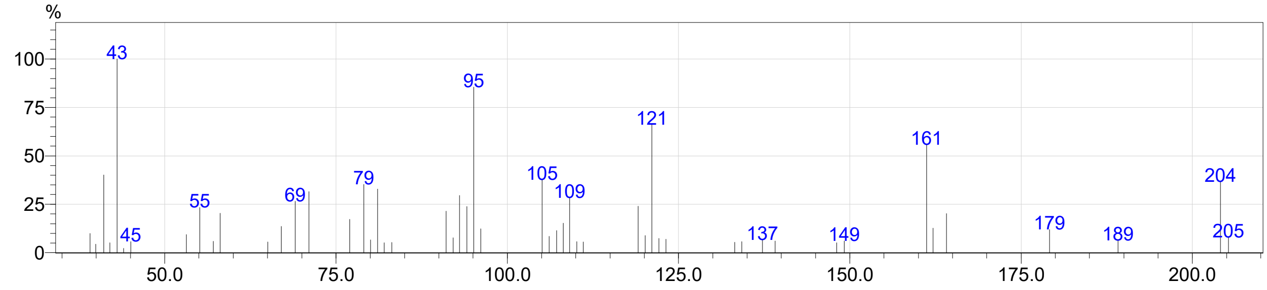

Figure S25. Mass spectrum of peak 24 (RT = 31.40 min).

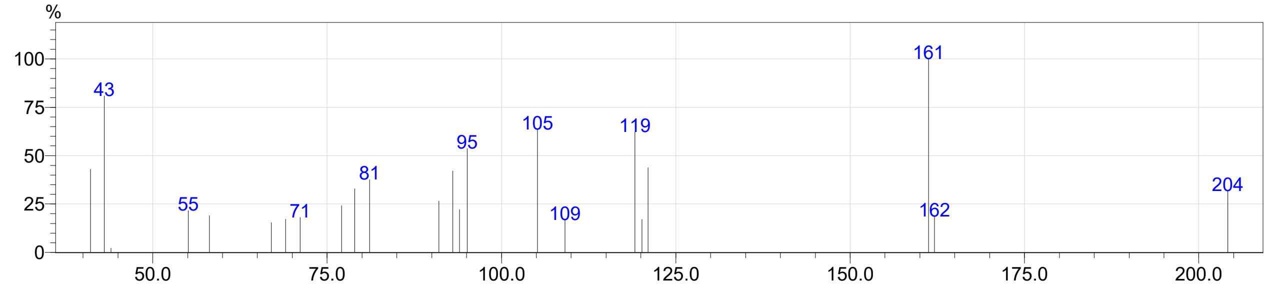

Figure S26. Mass spectrum of peak 25 (RT = 31.56 min).

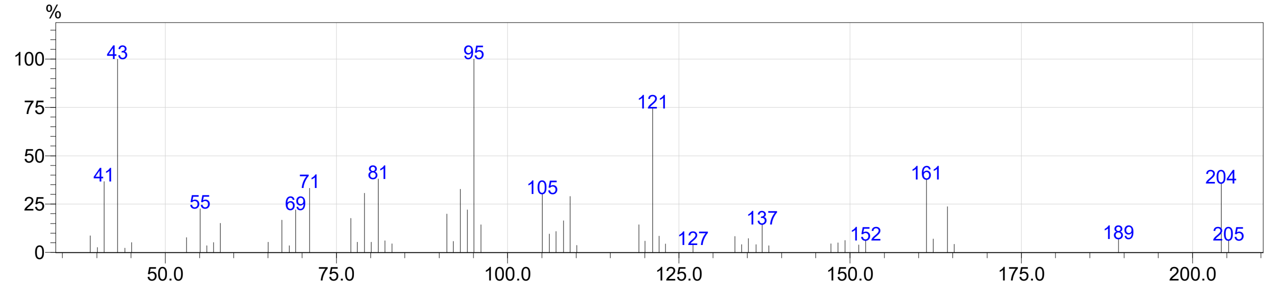

Figure S27. Mass spectrum of peak 26 (RT = 31.86 min).

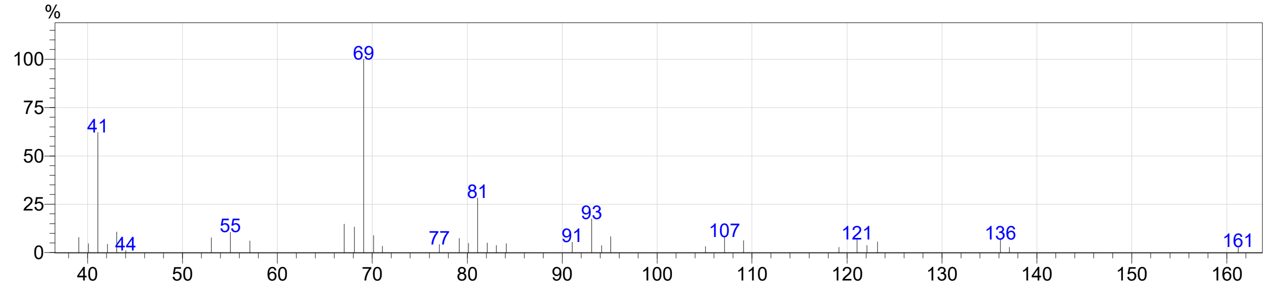

Figure S28. Mass spectrum of peak 27 (RT = 34.36 min).

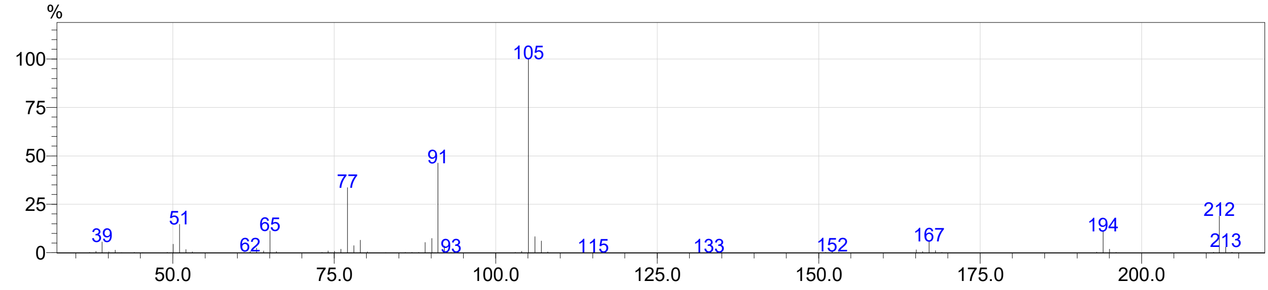

Figure S29. Mass spectrum of peak 28 (RT = 35.80 min).

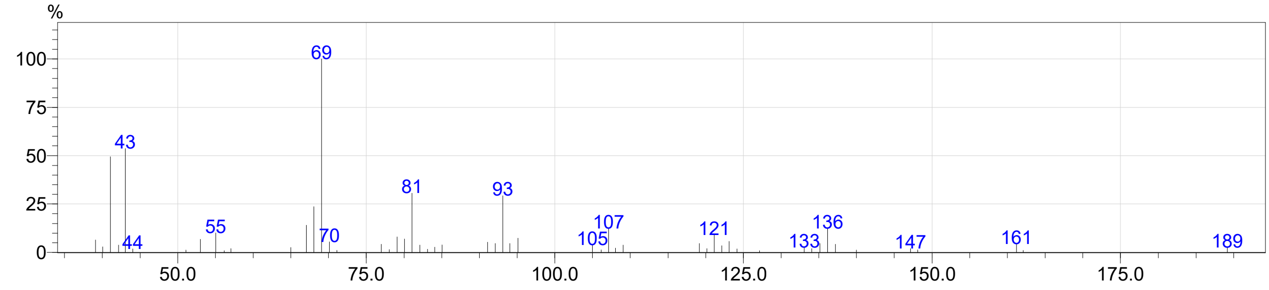

Figure S30. Mass spectrum of peak 29 (RT = 38.47 min).

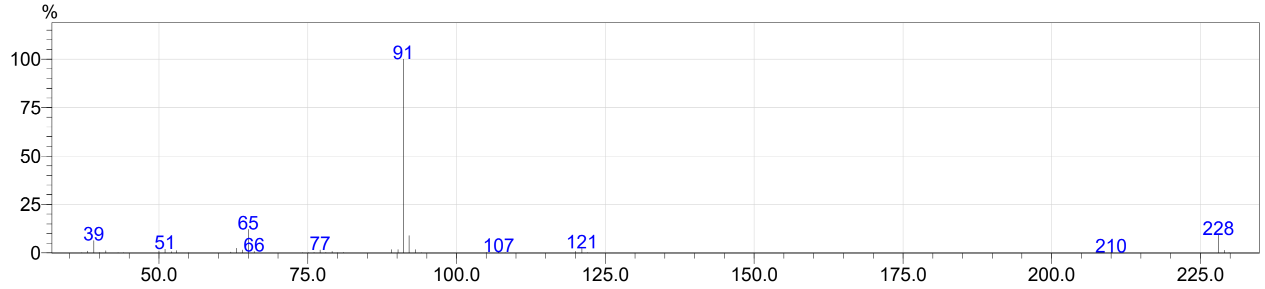

Figure S31. Mass spectrum of peak 30 (RT = 39.34 min).
